# Targeted Disruption of Lats1 and Lats2 in Mice Impairs Testis Development and Alters Somatic Cell Fate

**DOI:** 10.3390/ijms232113585

**Published:** 2022-11-05

**Authors:** Nour Abou Nader, Amélie Ménard, Adrien Levasseur, Guillaume St-Jean, Derek Boerboom, Gustavo Zamberlam, Alexandre Boyer

**Affiliations:** Centre de Recherche en Reproduction et Fertilité, Faculté de Médecine Vétérinaire, Université de Montréal, Saint-Hyacinthe, QC J2S 7C6, Canada

**Keywords:** Hippo signaling, Lats1/2, fetal Leydig cells, Sertoli cells, transgenic mouse model

## Abstract

Hippo signaling plays an essential role in the development of numerous tissues. Although it was previously shown that the transcriptional effectors of Hippo signaling Yes-associated protein (YAP) and transcriptional coactivator with PDZ-binding motif (TAZ) can fine-tune the regulation of sex differentiation genes in the testes, the role of Hippo signaling in testis development remains largely unknown. To further explore the role of Hippo signaling in the testes, we conditionally deleted the key Hippo kinases large tumor suppressor homolog kinases 1 and -2 (*Lats1* and *Lats2*, two kinases that antagonize YAP and TAZ transcriptional co-regulatory activity) in the somatic cells of the testes using an *Nr5a1*-cre strain (*Lats1*^flox/flox^;*Lats2*^flox/flox^;*Nr5a1*-cre). We report here that early stages of testis somatic cell differentiation were not affected in this model but progressive testis cord dysgenesis was observed starting at gestational day e14.5. Testis cord dysgenesis was further associated with the loss of polarity of the Sertoli cells and the loss of SOX9 expression but not WT1. In parallel with testis cord dysgenesis, a loss of steroidogenic gene expression associated with the appearance of myofibroblast-like cells in the interstitial space was also observed in mutant animals. Furthermore, the loss of YAP phosphorylation, the accumulation of nuclear TAZ (and YAP) in both the Sertoli and interstitial cell populations, and an increase in their transcriptional co-regulatory activity in the testes suggest that the observed phenotype could be attributed at least in part to YAP and TAZ. Taken together, our results suggest that Hippo signaling is required to maintain proper differentiation of testis somatic cells.

## 1. Introduction

In mice, testes are first derived from the intermediate mesoderm and arise from the proliferation of coelomic epithelial cells. Thickening of the coelomic epithelia initially form the adrenogonadal primordium (AGP) at embryonic day 9.5 (e9.5), a structure from which the adrenal cortex also develops [1,2]. Following primordial germ cell migration (around e10.5), the AGP separates into two distinct tissues as the adrenal progenitor cells migrate dorsomedially at e11.0 [3]. Shortly after, the sex determining region of chromosome Y (SRY) is first expressed in a subpopulation of somatic cells of the gonadal primordium (GP) [4,5] which induces their differentiation into Sertoli cells via a positive feedback loop between SRY (sex-determining region Y)-box 9 (SOX9) and fibroblast growth factor 9 (FGF9) [6,7,8]. After Sertoli cell differentiation, another subpopulation of somatic cells differentiates into fetal Leydig cells (FLCs) following the secretion of desert hedgehog (DHH) by the differentiating Sertoli cells and subsequent activation of Hedgehog signaling [9,10,11]. Tracing experiments either alone or in combination with single-cell RNAseq suggest that both Sertoli cells and FLCs originate from a common progenitor cell population [12,13,14]. Numerous factors, including Wilm’s tumor 1 (WT1) [15,16,17,18], nuclear receptor subfamily 5, group A, member 1 (NR5A1) [19], GATA binding protein 4 (GATA4) [20,21,22], insulin/insulin-like growth factor [23,24], Sine-oculis-related homeobox-1 and 4 (SIX1/4) [25], or Chromobox2 (Cbx2) [26,27], are involved in early stages of testicular somatic cell differentiation but additional factors and pathways are likely involved. 

Hippo is an evolutionarily conserved signaling pathway with well-established roles in cell fate determination and proliferation during embryonic development (reviewed in [28,29]). It consists of a kinase cascade that regulates two functionally redundant transcriptional co-activators, Yes-associated protein (YAP) and transcriptional coactivator with PDZ-binding motif (TAZ). In response to various extracellular signals, the mammalian STE20-like protein kinases 1 and -2 (MST1, MST2) are activated and phosphorylate the large tumor suppressor homolog kinases 1 and -2 (LATS1, LATS2), which in turn phosphorylate and inactivate YAP and TAZ [30,31,32,33]. When the cascade is inactivated, YAP and TAZ accumulate in the nucleus and interact principally with members of the TEA domain (TEAD) family of transcription factors to regulate the transcription of genes involved in cell growth, apoptosis, and proliferation [29,34]. 

Very little is known about the role of Hippo signaling in the testis, and most studies thus far have focused on its involvement in postnatal testicular physiology. Notably, it was shown that YAP is negatively regulated by FSH/PKA signaling in Sertoli cells [35,36] and that the number of YAP-positive Leydig cells increases in the mouse testis following chronic heat stress [37]. It was also shown that the loss of *Yap* and *Taz* decreases the expression of male sex-determining genes and increases the expression of female sex-determining genes in an immature Sertoli cell culture model [38], while the loss of *Lats1* and *Lats2* in granulosa cells have the opposite effect [39]. Finally, it was also demonstrated that the loss of Kindlin-2 (also known as fermitin family homolog 2; *Fermt2*) in Sertoli cells inhibits their proliferation and impairs cell–cell junction and blood–testis barrier maintenance by enhancing LATS1 interaction with YAP [40]. However, to this day, no studies have directly evaluated the role of LATS1 and LATS2, the core kinases of Hippo signaling, in testicular development. 

In this study, we generated a mouse model in which *Lats1* and *Lats2* were conditionally deleted in testicular somatic cells to characterize the role of the Hippo signaling pathway in the development of the testes. 

## 2. Results

### 2.1. Testicular Dysgenesis Is Observed following Concomitant Deletion of Lats1 and Lats2 in Testis Somatic Cells 

**Figure 1:**
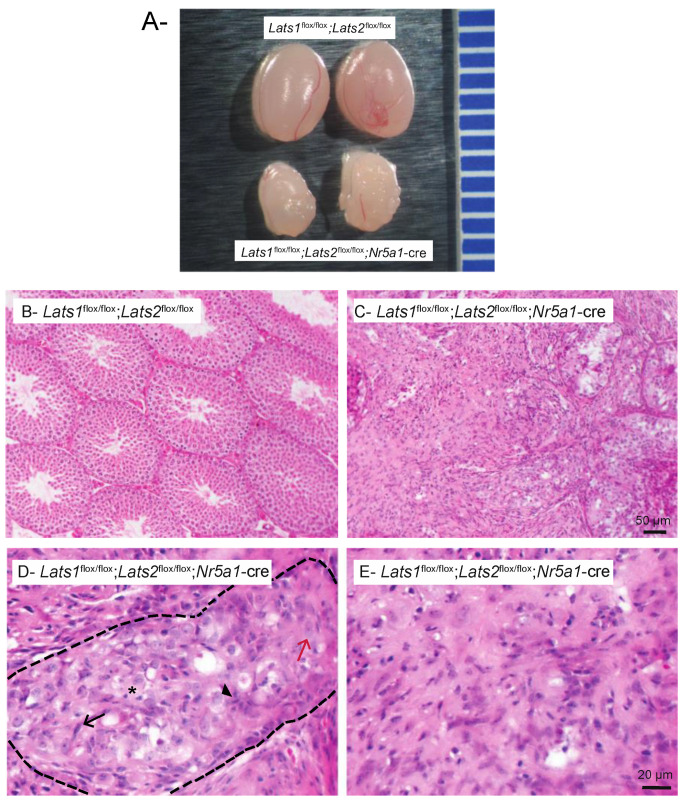
Testes of *Lats1*^flox/flox^;*Lats2*^flox/flox^;*Nr5a1*-cre mice are larger than controls and are histomorphologically abnormal. (**A**) Photographs of testes from 2-week-old *Lats1*^flox/flox^;*Lats2*^flox/flox^ (control) and *Lats1*^flox/flox^;*Lats2*^flox/flox^;*Nr5a1*-cre mice. Ruler graduations are in millimeters. (**B**,**C**) Photomicrographs comparing the testes of 2-week-old (**B**) *Lats1*^flox/flox^;*Lats2*^flox/flox^ and (**C**) *Lats1*^flox/flox^;*Lats2*^flox/flox^;*Nr5a1*-cre mice. (**D**,**E**) Photomicrographs illustrating the remaining seminiferous tubules (**D**) and interstitium (**E**) in *Lats1*^flox/flox^;*Lats2*^flox/flox^;*Nr5a1*-cre mice at higher magnification. Arrowhead = cells with a large nucleus and pale abundant cytoplasm. Red arrow = cell with an elongated large nucleus with an abundant cytoplasm. Black arrow = spindle-shaped cells. Asterisk = pyknotic cell. Dashes = delimitation of the seminiferous tubules. Scale bar in C is valid for B and scale bar in E is valid for D. Hematoxylin and eosin stain.

To investigate the role of Hippo signaling in testicular development, we genetically disrupted the core Hippo pathway kinases *Lats1*, *Lats2,* or both in testicular somatic cells. To do this, mice bearing floxed alleles for *Lats1* and/or *Lats2* were crossed with a *Nr5a1*-cre [41] strain that drives the expression of Cre recombinase in the precursors of both Sertoli cells and FLCs of the developing testes. *Lats1*^flox/flox^;*Nr5a1*-cre and *Lats2*^flox/flox^;*Nr5a1*-cre mice developed normally, were healthy, had normal lifespans, and were fertile. Testes from *Lats1*^flox/flox^; *Nr5a1*-cre and *Lats2*^flox/flox^; *Nr5a1*-cre mice appeared normal at the gross and histologic levels and were therefore not further studied. Conversely, double cKO (*Lats1*^flox/flox^;*Lats2*^flox/flox^;*Nr5a1*-cre) mice died between 2 and 3 weeks of age of adrenal failure due to Cre expression in the adrenal cortex [42]. Gross morphological assessment showed that the testes from 2-week-old *Lats1*^flox/flox^;*Lats2*^flox/flox^;*Nr5a1*-cre mice were smaller than the testes from the control *Lats1*^flox/flox^;*Lats2*^flox/flox^ animals and had a distinctive, lobular appearance (Figure 1A). 

Histological analyses revealed striking abnormalities in the testes of *Lats1*^flox/flox^;*Lats2*^flox/flox^;*Nr5a1*-cre mice. Seminiferous tubules were rarely observed and were very large, with disorganized cells and no lumen (Figure 1B–E). Several different types of cells were present within these tubules, with some cells having elongated large nuclei with an abundant cytoplasm, others having round large nuclei and abundant pale cytoplasm, others having a spindle shape, and others being pyknotic (Figure 1D). Furthermore, the tubules were surrounded by spindle-shaped interstitial cells reminiscent of fibroblasts or myofibroblasts (Figure 1E). 

### 2.2. Testicular Dysgenesis Is First Observed at e14.5 in Lats1^flox/flox^;Lats2^flox/flox^;Nr5a1-cre Mice

**Figure 2:**
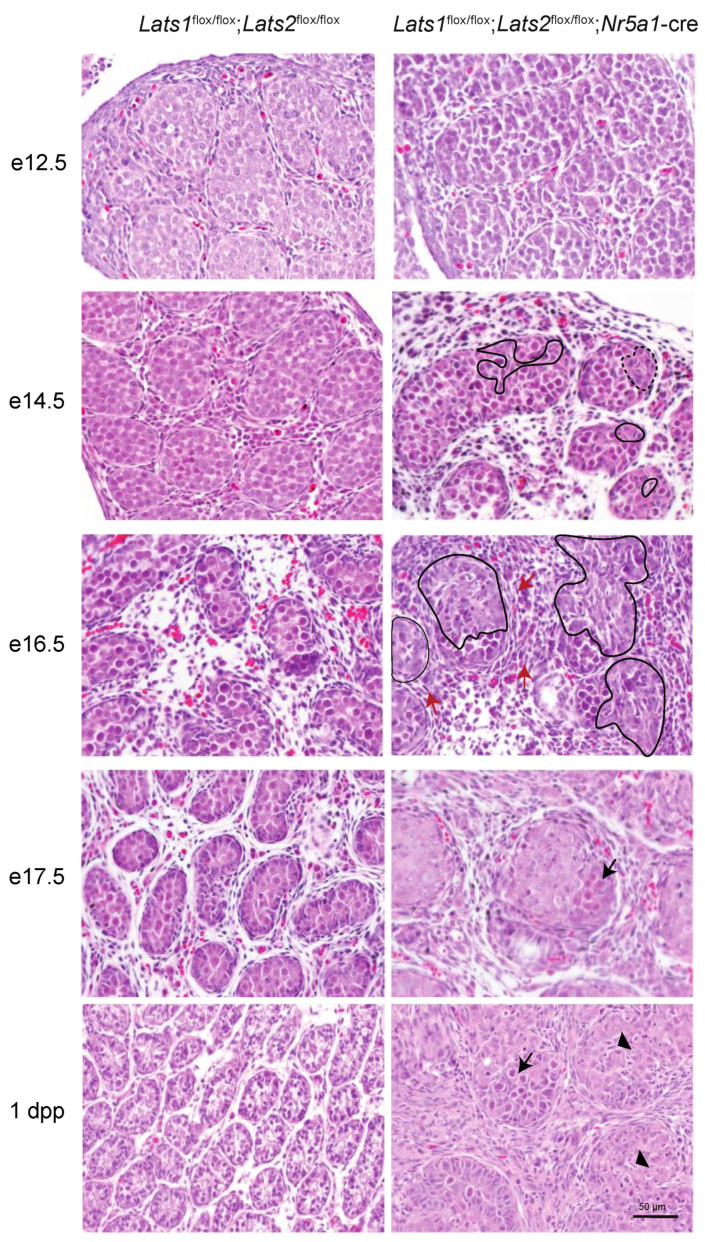
Progressive testicular dysgenesis in the *Lats1*^flox/flox^;*Lats2*^flox/flox^;*Nr5a1*-cre mice. Photomicrographs comparing testis histology of *Lats1*^flox/flox^;*Lats2*^flox/flox^;*Nr5a1*-cre with that of *Lats1*^flox/flox^;*Lats2*^flox/flox^ controls during development. Dashed lines = testis cords dysgenesis, black arrow = germ cells, red arrow = interstitial spindle-shaped cells, arrowhead = spindle-shaped cells in the testis cords. Scale bar (lower right) is valid for all images. Hematoxylin and eosin stain.

To analyze the onset and evolution of the phenotype observed in the testes of *Lats1*^flox/flox^;*Lats2*^flox/flox^;*Nr5a1*-cre mice, embryos were collected from embryonic days e12.5 onward, and histopathologic examination of the developing testes was performed. These analyses showed that testes from e12.5 *Lats1*^flox/flox^;*Lats2*^flox/flox^;*Nr5a1*-cre mice were phenotypically indistinguishable from age-matched controls (Figure 2) (*n* = 6). However, starting at e14.5, dysgenesis of a few testis cords became apparent in 30% of the mutant animals (*n* = 10) (Figure 2, dashed lines), while no abnormal phenotype was apparent in the remaining animals (Figure S1A). By e16.5, dysgenesis was apparent in a larger portion of the testis cords of all mutant animals (*n* = 4) and an accumulation of spindle-shaped cells was apparent in some regions of the interstitium (Figure 2, red arrow). At e17.5, the phenotypic changes observed in the testes of *Lats1*^flox/flox^;*Lats2*^flox/flox^;*Nr5a1*-cre mice were exacerbated and dysgenesis was observed in all of the testis cords, with the Sertoli cells being disorganized and having a large prominent cytoplasm and the remaining germ cells being mostly apoptotic (Figure S2A, arrowhead) and pushed over to one side of the cords (*n* = 10) (Figure 2, arrow, Figure S2B). Testis cords were also larger than their control counterparts and the interstitium was mostly occupied by spindle-shaped cells (Figure 2). Phenotypic changes observed in the testes of *Lats1*^flox/flox^;*Lats2*^flox/flox^;*Nr5a1*-cre mice were even more pervasive and pronounced in testes of 1dpp mutant animals (Figure 2), with germ cells being absent in the majority of the tubules (Figure 2, Figure S2B), the presence of some spindle-shaped cells of indeterminate origin located inside the testis cords (Figure 2, arrowhead), and the interstitium being completely occupied by spindle-shaped cells. 

### 2.3. Hippo Signaling Is Inactivated in Late Stages of Testicular Differentiation

**Figure 3:**
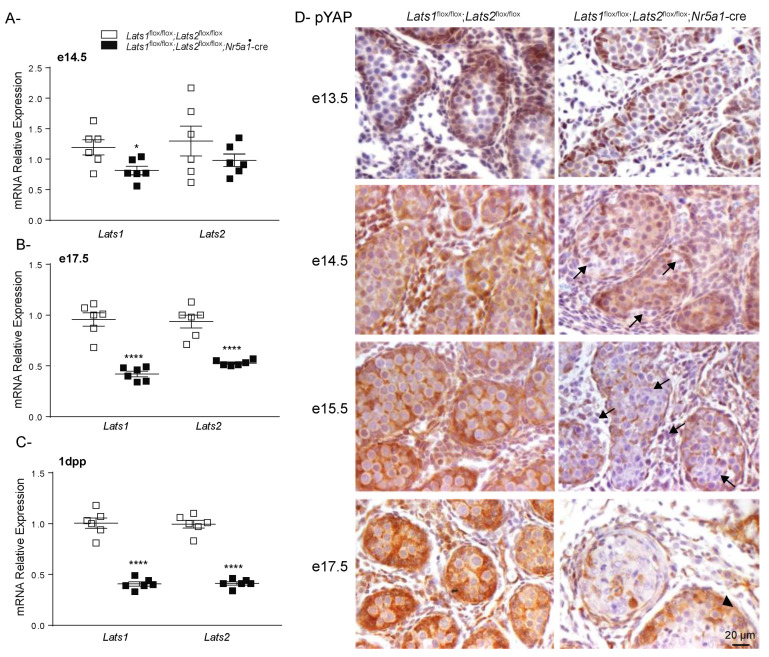
Efficiency of *Lats1* and *Lats2* knockdown in the testes of *Lats1*^flox/flox^;*Lats2*^flox/flox^;*Nr5a1*-cre mice. (**A**–**C**) RT-qPCR analysis of *Lats1* and *Lats2* mRNA levels in the testes of (**A**) e14.5, (**B**) e17.5, and (**C**) 1 dpp mice of the indicated genotypes (*n* = 6 animals/genotype). All data were normalized to the housekeeping gene *Rpl19* and are expressed as means (columns) ± SEM (error bars). Asterisks = significantly different from control (* *p* < 0.05; **** *p* < 0.0001). (**D**) Immunohistochemical analysis of phospho-YAP expression in the testes of mice of the indicated genotypes. Scale bar (lower right) is valid for all images. Arrowhead = cells with inefficient recombination. Arrow = cells with efficient recombination.

Although the *Nr5a1* promoter is active from the early stages of adrenal–gonadal primordium formation at around e9.0–e10 in mice [43], the phenotypic changes observed in the testes of *Lats1*^flox/flox^;*Lats2*^flox/flox^;*Nr5a1*-cre mice were not apparent until e14.5. This is later than expected but is consistent with the timing of phenotypic changes and inactivation of *Lats1* and *Lats2* observed in the developing adrenal glands of mutant animals, which is also first observed at e14.5 [42]. To determine if this was also the case in the testes, RT-qPCR analyses were performed on developing testes at embryonic days e14.5, e17.5 and 1dpp. The results showed a small but significant reduction (33%) of testis *Lats1* mRNA levels (but no reduction of *Last2* mRNA levels) in e14.5 mutant animals (Figure 3A); 56% and 42% decreases in testis *Lats1* and *Lats2* mRNA levels in e17.5 mutant animals (Figure 3B); and 60% and 58% decreases in testis *Lats1* and *Lats2* mRNA levels in 1dpp mutant animals (Figure 3C). 

We were unable to obtain quality immunohistochemistry for LATS1/2 to complement our RT-qPCR *Lats1/2* expression data. Loss of *Lats1* and *Lats2* was therefore assessed indirectly by evaluating the phosphorylation of their substrate YAP. At e13.5, phospho-YAP was readily detected in the nucleus of the Sertoli cells of both control and mutant testes (Figure 3D). Weak expression of phospho-YAP could also be detected in some interstitial cells of both control and mutant testes. These results suggest that Hippo signaling was not inactivated in mutant animals at this time point. At e14.5, phospho-YAP expression was readily detected in the Sertoli cells and interstitial cells of the control testes (Figure 3D) and some mutant animals (Figure S1B). However, a decrease in the expression of phospho-YAP could be observed in a few Sertoli cells of some mutant animals (Figure 3D, Arrow). Furthermore, phospho-YAP expression remained weak and mostly cytoplasmic in the interstitium of these animals (Figure 3D). At e15.5 and e17.5, phospho-YAP expression remained elevated in both the interstitial cells and the Sertoli cells of control testes (Figure 3D). However, in the testes of the mutant animals, phospho-YAP was no longer detected in the majority of the interstitial cells (Figure 3D). Phospho-YAP expression was also lost in the majority (but not all) of the Sertoli cells, suggesting that recombination had not yet occurred in all Sertoli cells (Figure 3D, arrowhead). The pattern of expression of phospho-YAP is in accordance with the loss of *Lats1* and *Lats2* in the mutant animals and confirmed that inactivation of Hippo signaling occurs in both the FLCs and Sertoli cells. Variation between phospho-YAP expression in e14.5 mutant animals combined with the RT-qPCR data also suggests that recombination is initiated around that time point in mutant animals.

**Figure 4:**
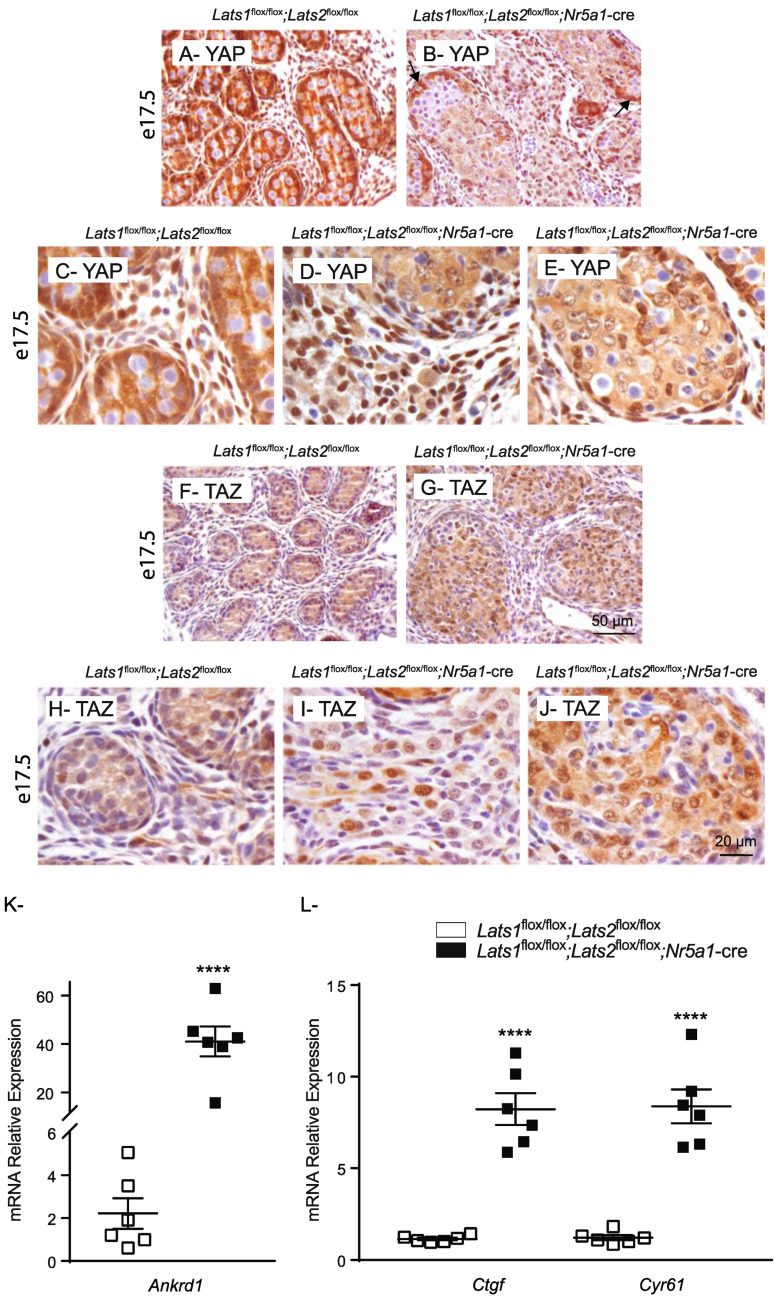
YAP/TAZ activity increases in the testes of *Lats1*^flox/flox^;*Lats2*^flox/flox^;*Nr5a1*^cre/+^ mice. (**A**–**E**) Immunohistochemical analysis of YAP expression in testes of e17.5 mice of the indicated genotypes. (**F**–**J**) Immunohistochemical analysis of TAZ expression in testes of e17.5 mice of the indicated genotypes. Arrow = YAP expression in non-recombined Sertoli cells. Scale bar in (**G**) is valid for (**A**,**B**,**F**). Scale bar in (**J**) is valid for (**C**–**E**,**H**,**I**). (**K**,**L**) RT-qPCR analysis of YAP/TAZ downstream targets mRNA levels in the testes of e17.5 mice of the indicated genotypes (*n* = 6 animals/genotype). All data were normalized to the housekeeping gene *Rpl19* and are expressed as means (columns) ± SEM (error bars). Asterisks = significantly different from control (**** *p* < 0.0001).

Since the inactivation of Hippo signaling is also normally associated with an increase in the nuclear localization of both YAP and TAZ, the expression of YAP and TAZ and their classic downstream targets were further evaluated at e17.5 by immunohistochemistry (using antibodies that mark both their phosphorylated and unphosphorylated forms). At this time point, YAP expression was detected in the cytoplasm and nucleus of most interstitial cells and in the Sertoli cells of control animals (Figure 4A,C). In the testes of mutant animals, expression of YAP was observed in the nucleus of most interstitial cells (Figure 4B,D). Nuclear, but generally weak, expression of YAP was detected in the Sertoli cells located in the middle of the tubules (Figure 4B,E) while strong YAP expression was observed in the Sertoli cells located at the periphery of some tubules (Figure 4B, arrow). However, this last population also expressed phospho-YAP (Figure 3D), suggesting that they correspond to Sertoli cells in which recombination did not occur. Weak TAZ expression was detected in the cytoplasm of the majority of Sertoli cells and some interstitial cells, as well as in the nucleus of rare interstitial cells in the control animals (Figure 4F,H). However, nuclear expression of TAZ was detected in a larger proportion of interstitial cells in the testes of *Lats1*^flox/flox^;*Lats2*^flox/flox^;*Nr5a1*-cre mice (Figure 4G,I). Furthermore, TAZ expression was detected in both the cytoplasm and nucleus of the recombined Sertoli cells (Figure 4G,J), suggesting that the increase of TAZ activity might play a greater role in the observed phenotype.

To further determine if the transcriptional co-regulatory activity of YAP and TAZ was increased in *Lats1*^flox/flox^;*Lats2*^flox/flox^;*Nr5a1*-cre mice, the mRNA levels of the well-established YAP/TAZ target genes ankyrin repeat domain 1 (*Ankrd1*), connective tissue growth factor (*Ctgf*), and cysteine-rich and angiogenic inducer 61 (*Cyr61*) were quantified by RT-qPCR. A 40-fold increase in the mRNA levels of *Ankrd1* and an 8-fold increase in the mRNA levels of *Ctgf* and *Cyr61* were observed in the testes of e17.5 *Lats1*^flox/flox^;*Lats2*^flox/flox^;*Nr5a1*-cre mice (Figure 4K), confirming that the expression of known targets of YAP and TAZ is increased in the testes of mutant animals. Taken together, these data suggest that transcriptional regulatory activity of YAP and/or TAZ is increased in the somatic cells of *Lats1*^flox/flox^;*Lats2*^flox/flox^;*Nr5a1*-cre mice, and most likely play a role in the observed phenotypic changes.

### 2.4. Disruption of Lats1 and Lats2 Alters the Epithelization and the Differentiation of the Sertoli Cells during Testis Development 

**Figure 5:**
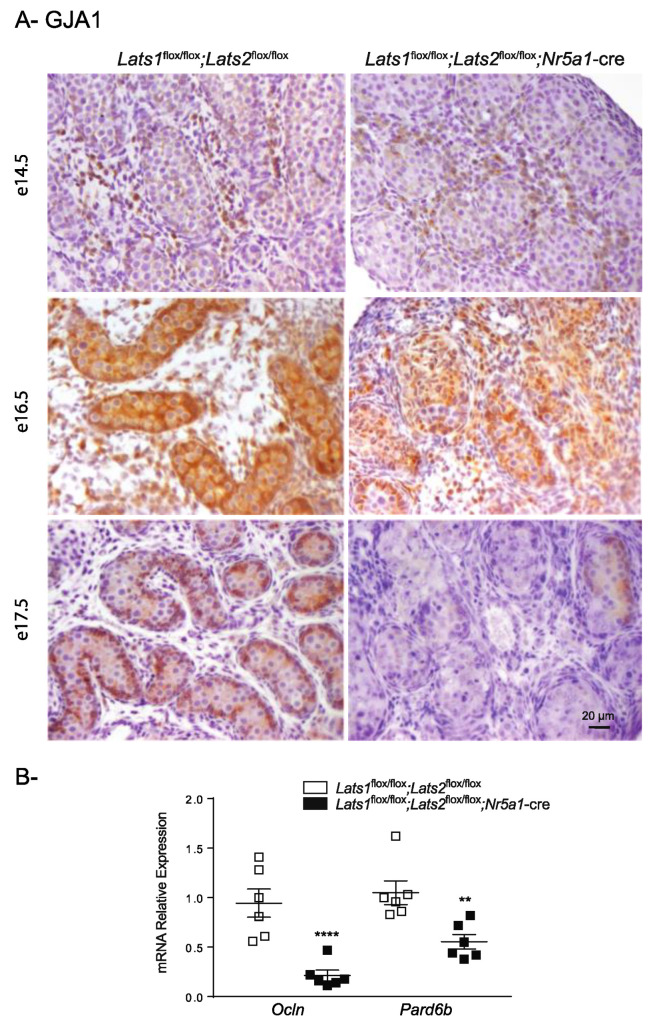
Loss of *Lats1* and *Lats2* affects the polarity of the Sertoli cells. (**A**) Immunohistochemical analysis of GJA1 expression in testes of mice of the indicated genotypes. Scale bar (lower right) is valid for all images. (**B**) RT-qPCR analysis of *Ocln* and *Pard6b* mRNA levels in the testes of 1 dpp mice of the indicated genotypes (*n* = 6 animals/genotype). All data were normalized to the housekeeping gene *Rpl19* and are expressed as means (columns) ± SEM (error bars). Asterisks = significantly different from control (** *p* < 0.01; **** *p* < 0.0001).

YAP and TAZ activation have been known to play key roles in processes such as epithelial-to-mesenchymal (EMT) transition and fibrosis, two cellular processes that share many molecular characteristics [44,45,46]. Misexpression of mesenchymal-associated pathways could explain both the loss polarity of Sertoli cells and the appearance of fibroblast/myofibroblast-like cells in the interstitial tissue. To determine if normal epithelization of the Sertoli cells was affected in the testes of mutant animals, markers of Sertoli cell junctions were then evaluated. Expression of GAP junction protein alpha-1 (GJA1), (an important component of Sertoli/Sertoli interactions [47,48]) expression was first analyzed. At e14.5, expression of GJA1 was marginal in both the control and mutant animals, with most of the expression detected in the interstitial tissue and only weak expression observed in the testis cords (Figure 5A). A marked increase in the expression of GJA1 was observed in the testis cords of control and mutant animals at e16.5. However, GJA1 expression was more important at the periphery of the testis cords in control animals, a pattern of expression that is not observed in mutant animals (Figure 5A). At e17.5, GJA1 expression was mainly observed at the periphery of testis cords (at the presumed Sertoli cell junctions), while GJA1 expression was lost in the majority of the testis cords of the mutant animals (Figure 5A). Furthermore, the mRNA levels for occludin (*Ocln*), a major contributor to Sertoli cell tight junctions, and for par-6 family cell polarity regulator beta (*Pard6b*), a gene important for apicobasal polarity, also decreased in the testes of mutant animals (Figure 5B) suggesting that epithelization of the Sertoli cells was affected in *Lats1*^flox/flox^;*Lats2*^flox/flox^;*Nr5a1*-cre mice.

**Figure 6:**
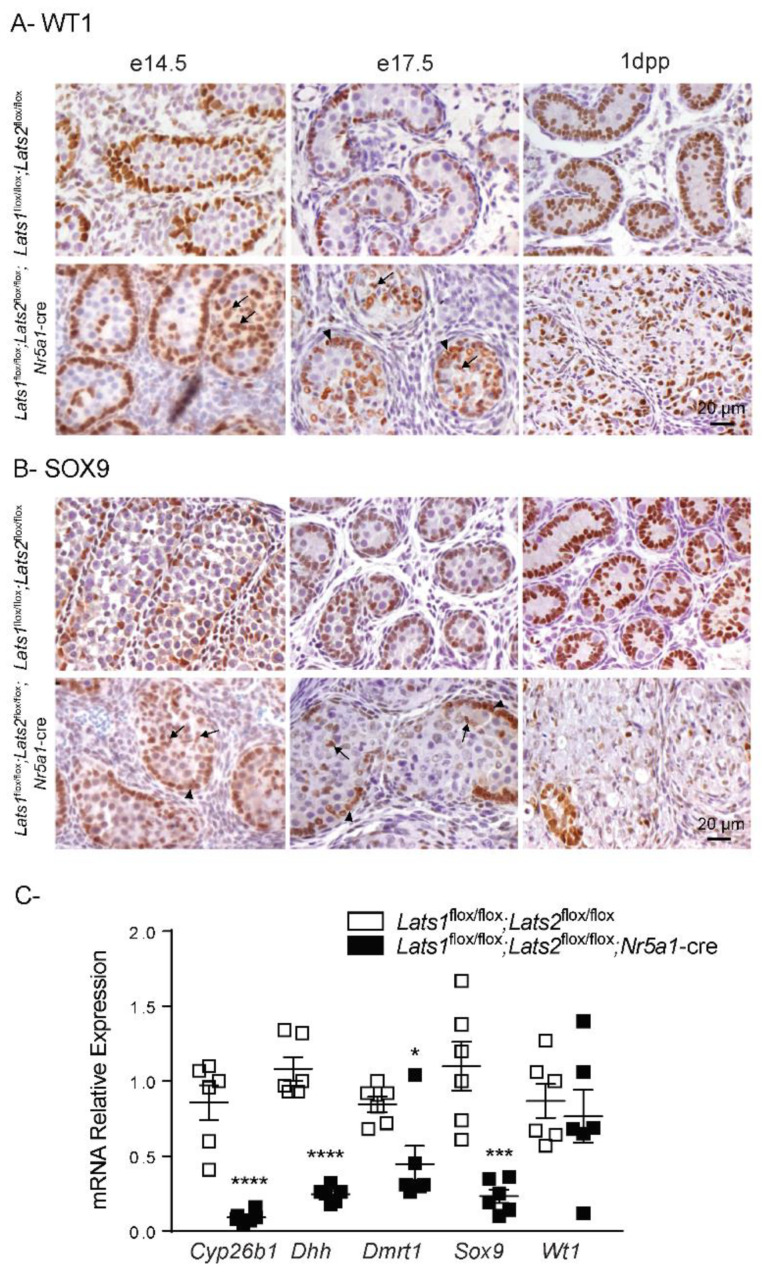
Loss of *Lats1* and *Lats2* affects expression of key Sertoli cell markers. (**A**) Immunohistochemical analysis of WT1 expression in testes of mice of the indicated genotypes. (**B**) Immunohistochemical analysis of SOX9 expression in testes of mice of the indicated genotypes. Arrow = immunopositive cells that lost their polarity. Arrowhead = immunopositive cells with normal polarity. Scale bar (lower right) is valid for all images. (**C**) RT-qPCR analysis of Sertoli cell markers in testes of 1dpp mice of the indicated genotypes (*n* = 6 animals/genotype). All data were normalized to the housekeeping gene *Rpl19* and are expressed as means (columns) ± SEM (error bars). Asterisks = significantly different from control (* *p* < 0.05; *** *p* < 0.001; **** *p* < 0.0001).

To further determine if the loss of *Lats1* and *Lats2* in Sertoli cells affects their cellular identity, immunohistochemistry for WT1 and SOX9 was performed. In control animals, WT1+ and SOX9+ positive cells were mostly located at the periphery of the testis cords from e14.5 to 1dpp, though rare positive cells were observed in the middle of the cords at e14.5 (Figure 6A,B). In the testes of mutant animals, an increasing portion of WT1+ was found in the middle of the testis cords between e14.5 and 1dpp (Figure 6A, arrow), again suggesting that these Sertoli cells had lost their polarity. Interestingly, even if localization of SOX9+ cells were initially similar to WT1+ cells in *Lats1*^flox/flox^;*Lats2*^flox/flox^;*Nr5a1*-cre, only rare SOX9+ cells could be detected in the seminiferous tubules of 1dpp mice (Figure 6B). To confirm the results obtained by immunohistochemistry and to further characterize the Sertoli cells in *Lats1*^flox/flox^;*Lats2*^flox/flox^;*Nr5a1*-cre mice, the expression of the Sertoli cell markers cytochrome P450, 26, retinoic acid B1 (*Cyp26b1*), desert hedgehog (*Dhh*), doublesex and mab-3-related transcription factor 1 (*Dmrt1*), *Sox9,* and *Wt1* were evaluated by RT-qPCR at 1dpp. Interestingly, the mRNA levels of all Sertoli cell markers decreased significantly except for the mRNA levels of *Wt1,* which were maintained in the testes of mutant animals (Figure 6C). To determine if Sertoli cells could differentiate into granulosa cells, RT-qPCR were also performed for forkhead box L2 (*Foxl2*) and wingless-type MMTV integration site family, member 4 (*Wnt4*). However, expression levels of both markers did not increase in the testes of mutant animals (Figure S3). Taken together, the epithelization defect and the expression of Sertoli cell markers suggest that the loss of *Lats1/2* affects the identity of the Sertoli cells but that these cells do not transdifferentiate into granulosa cells.

### 2.5. Disruption of Lats1 and Lats2 Causes the Loss of Steroidogenic Markers and Leads to Fibrosis of the Testis Interstitium 

**Figure 7:**
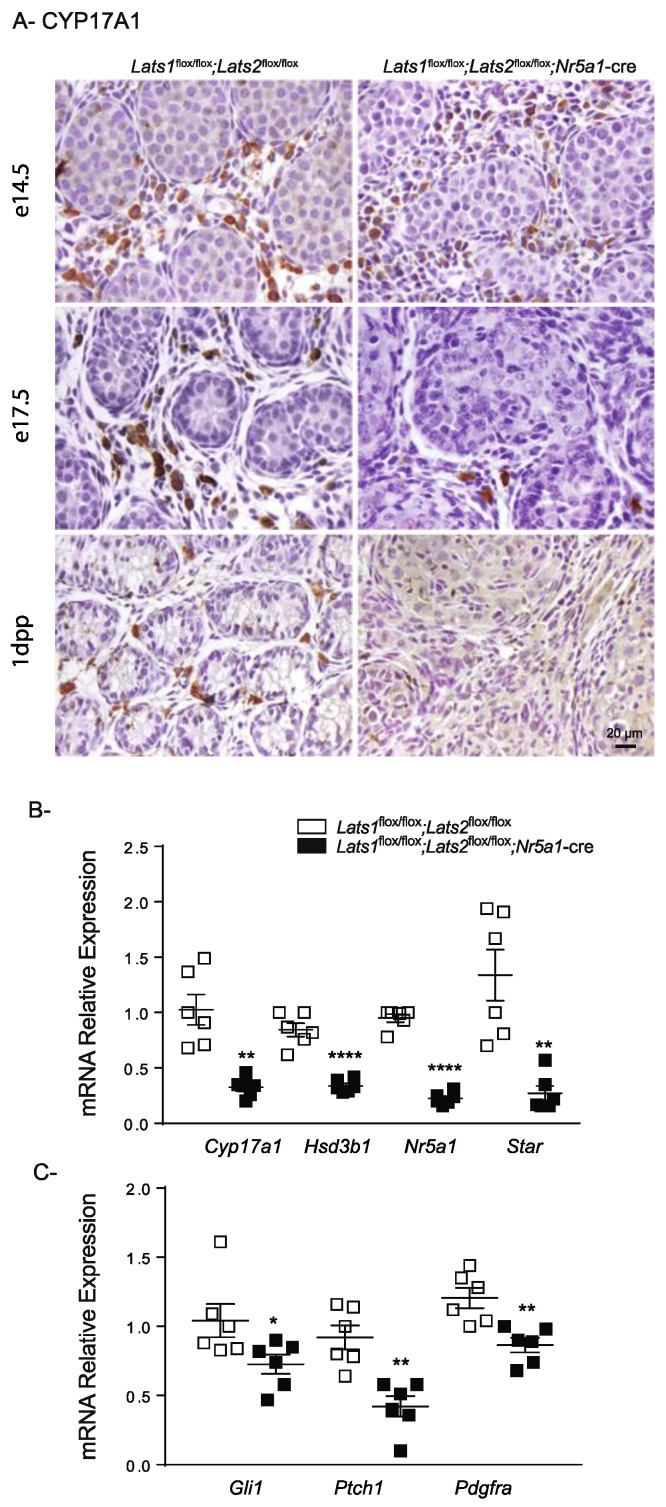
Loss of *Lats1* and *Lats2* affects FLCs. (**A**) Immunohistochemical analysis of CYP17A1 expression in testes of mice of the indicated genotypes. Scale bar (lower right) is valid for all images. (**B**,**C**) RT-qPCR analysis of steroidogenesis (**B**) and progenitor FLC markers (**C**) in testes of 1dpp mice of the indicated genotypes (*n* = 6 animals/genotype). All data were normalized to the housekeeping gene *Rpl19* and are expressed as means (columns) ± SEM (error bars). Asterisks = significantly different from control (* *p* < 0.05; ** *p* < 0.01; **** *p* < 0.0001).

To characterize the consequences of *Lats1* and *Lats2* deletion in FLCs, CYP17A1 expression was evaluated by immunohistochemistry. At e14.5, the expression of CYP17A1 was indistinguishable between control and mutant animals (Figure 7A). However, at e17.5, the number of CYP17A1+ cells was considerably reduced in the testes of mutant animals compared to their control counterparts (Figure 7A).

By 1dpp, CYP17A1+ cells were completely absent in the testes of mutant animals (Figure 7A). To confirm the loss of steroidogenic cells, *Cyp17a1*, hydroxy-delta-5-steroid dehydrogenase, 3 beta- and steroid-delta-isomerase 1 (*Hsd3b1*), nuclear receptor subfamily 5, group A, member 1 (*Nr5a1*) and steroidogenic acute regulatory protein (*Star*) mRNA levels were evaluated. As expected, a marked loss of every steroidogenic marker was observed in testes of *Lats1*^flox/flox^;*Lats2*^flox/flox^;*Nr5a1*-cre mice (Figure 7B). Together, these results suggest that like Sertoli cells, FLC differentiation initially occurs but that FLC subsequently lose their steroidogenic capacity. 

To further characterize the FLC population, expression of platelet-derived growth factor receptor, alpha polypeptide (*Pdgfra*), and patched homolog 1 (*Ptch1*), which are initially expressed in both interstitial cells and non-steroidogenic FLCs progenitors [49,50,51,52], as well as the effector of Hedgehog signaling *Gli1,* were also evaluated by RT-qPCR at 1dpp. Again, a significant decrease in the mRNA levels of all three markers were observed in testes of *Lats1*^flox/flox^;*Lats2*^flox/flox^;*Nr5a1*-cre mice (Figure 7C), arguing against the possibility that FLCs dedifferentiate into non-steroidogenic FLCs progenitors. 

**Figure 8:**
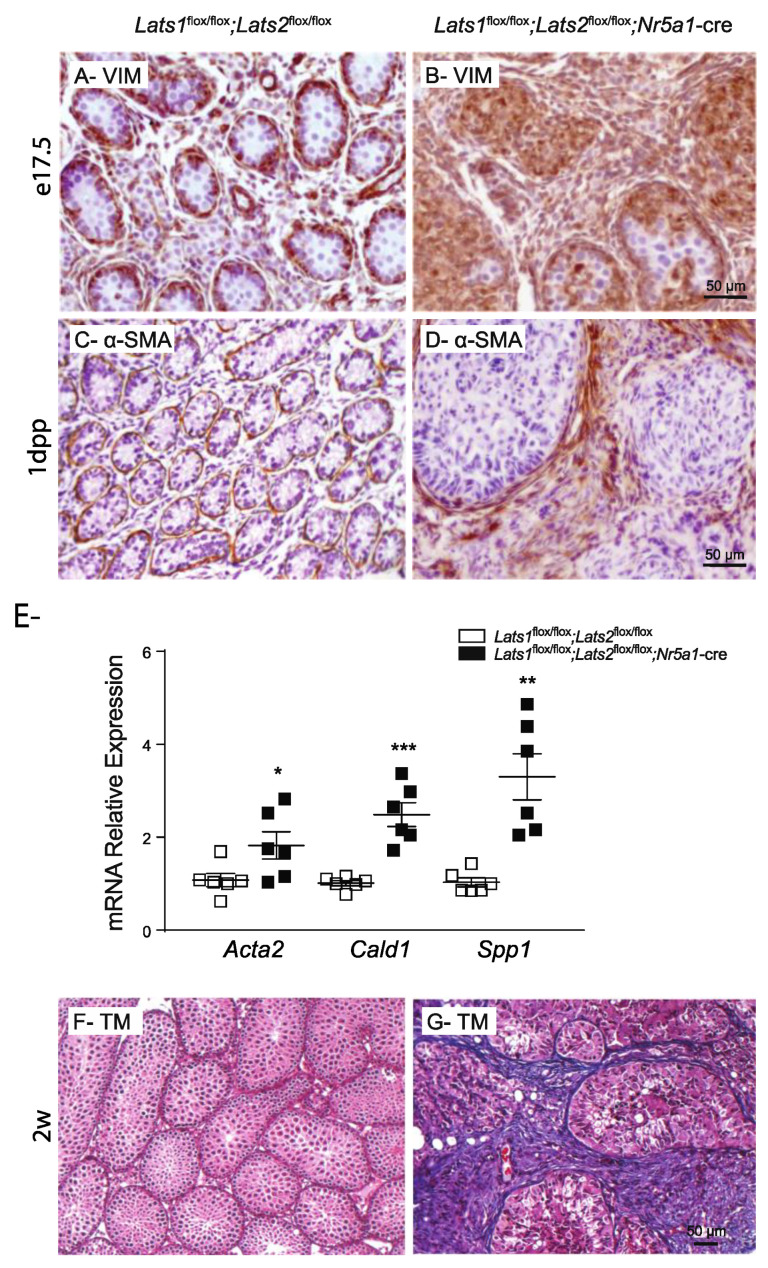
Loss of *Lats1* and *Lats2* leads to fibrosis in the testis interstitial tissue. (**A**,**B**) Immunohistochemical analysis of VIM expression in testes of e17.5 mice of the indicated genotypes. (**C**,**D**) Immunohistochemical analysis of α-SMA expression in testes of 1dpp mice of the indicated genotypes. (**E**) RT-qPCR analysis of myofibroblast markers in testes of 1dpp mice of the indicated genotypes (*n* = 6 animals/genotype). All data were normalized to the housekeeping gene *Rpl19* and are expressed as means (columns) ± SEM (error bars). Asterisks = significantly different from control (* *p* < 0.05; ** *p* < 0.01; *** *p* < 0.001). (**F**,**G**) Masson’s trichrome staining of 2-week-old mice of the indicated phenotype. Scale bars in the right panels are valid for the left panels.

The progressive loss of steroidogenesis and the adoption of a myofibroblast-like spindle shape by the testis interstitial cells was reminiscent of similar changes that occur in the adrenal gland of *Lats1*^flox/flox^;*Lats2*^flox/flox^;*Nr5a1*-cre mice [42]. In the latter, adrenocortical cells (which share a common embryonic origin with FLCs) overexpress the mesenchymal cell and myofibroblast marker vimentin (VIM), followed by an increase in the expression of the myocyte and myofibroblast marker α-SMA [42]. To determine if this was also the case in the testes, VIM and α-SMA expression were evaluated in the testes of control and mutant mice. A marked increase in the number of VIM+ cells was detected in the interstitial tissue of the mutant testes compared to controls (Figure 8A,B) at e17.5. Interestingly, the expression pattern of VIM was also modified in the Sertoli cells (Figure 8A,B). An increase in α-SMA expression was detected in the majority of the interstitial cells of mutant animals (Figure 8C,D) but only at 1dpp. This increase of expression was also associated with an increase in the mRNA levels of actin, alpha 2, smooth muscle, aorta (*Acta2*, the gene coding for α-SMA), and of the myofibroblast markers caldesmon 1 (*Cald1*) and secreted phosphoprotein 1 (*Spp1*) (Figure 8E), as well as with the eventual accumulation of collagen fibers in the interstitial tissue of older animals (Figure 8F, G). Together these results suggest that the transdifferentiation of the FLCs cells could be at least in part responsible for the interstitial fibrosis. 

## 3. Discussion

In recent years, Hippo has been identified as one of the most important signaling pathways involved in tissue development; however, no study has evaluated its function in the development of the testes. We report herein that the concomitant inactivation of the two core kinases of the Hippo pathway, *Lats1* and *Lats2*, alters the fate of the testicular somatic cells.

Even though NR5A1 is normally expressed early during gonadal development (e9.5–10.0) in a progenitor cell population that differentiate into both Sertoli cells and FLCs [13], the loss of *Lats1/2* observed in the *Lats1*^flox/flox^;*Lats2*^flox/flox^;*Nr5a1*-cre model was only apparent in late stages of testis development, as suggested by the loss of *Lats1* and *Lats2* mRNA levels and concomitant loss of YAP phosphorylation. This result could be explained in one of two ways. First, it is possible that recombination of the *Lats1* and *Lats2* floxed alleles is not efficient in testis somatic cells. Second, it is possible that the *Nr5a1*-cre model used in the present study does not sufficiently express Cre in the early stages of testis development to allow proper recombination. In that regard, it is important to note that the *Nr5a1*-cre model used for this study was the one developed by the Lowell group [41], which has never been fully characterized in the early stages of testis development and which has been used more sparingly than the model developed by the Parker group [53]. No matter the reason, our results suggest that efficient recombination only occurs when Sertoli cells and FLCs are already committed to their respective lineage, and it can be concluded that the phenotypic changes observed in the mutants herein are most likely due to the transdifferentiation of the Sertoli cells and FLCs rather than to the altered differentiation of their common progenitors. 

Inactivation of *Lats1/2* seems to impair the development of both Sertoli cells and FLCs cells in the *Lats1*^flox/flox^;*Lats2*^flox/flox^;*Nr5a1*-cre animals. In Sertoli cells, the redistribution/increase of VIM expression, the absence of increase in junction protein, alpha 1, 43 KDA (GJA1) expression, the decrease in the expression of *Ocln* and *Pard6b,* and the presence of WT1+ and SOX9+ cells at the center of the tubules together suggest that Sertoli cells lose their epithelial nature and potentially gain characteristics of mesenchymal cells in mutant mice. Epithelial–mesenchymal transition (EMT) is a common event following the downregulation of Hippo signaling and the increase in YAP/TAZ activity [39,54,55,56,57,58,59] and has notably been reported to occur in ovarian granulosa cells [39], which share a common precursor with Sertoli cells [4]. Interestingly, it was shown that YAP and WT1 act in synergy to loosen cell–cell contacts and trigger EMT in the epithelial MDCK cell line [59], suggesting that YAP or TAZ could act with WT1 in a similar manner in Sertoli cells. It was also previously observed that Sertoli cells initially have mesenchyme-like behaviors and morphology before migrating to the periphery of the testis cords and gaining characteristics of epithelial cells [60]. The phenotype observed in the *Lats1*^flox/flox^;*Lats2*^flox/flox^;*Nr5a1*-cre model could therefore suggest that the Sertoli cells regain characteristics of mesenchymal cells and dedifferentiate to a more primitive stage of development. The fact that WT1 expression (whose expression precedes the differentiation of the Sertoli cell expression [16,61,62]) is maintained in the majority of the presumptive Sertoli cells while SOX9 and *Dhh* are lost also argues in favor of the dedifferentiation of the Sertoli cells. Further experiments are required to delineate the role of Hippo signaling in the maintenance of the identity of the Sertoli cells.

Loss of SOX9 expression in the presumptive immature Sertoli cells of the *Lats1*^flox/flox^;*Lats2*^flox/flox^;*Nr5a1*-cre animals was surprising as it was previously shown that loss of *Yap* and *Taz* leads to the downregulation of SOX9 expression in primary cultures of immature Sertoli cells [38] and that loss of *Lats1* and *Lats2* in postnatal granulosa cells leads to an increase in SOX9 expression and the appearance of Sertoli-like cells in the ovary [39]. This finding suggests that interactions between Hippo signaling and *Sox9* expression are more complex than expected. Since YAP and TAZ can bind several transcription factors, it is possible that the presence or absence of other transcription factors during different stages of Sertoli cell development dictate the regulation of *Sox9* by Hippo signaling. Another possibility is that very tight regulation of the transcriptional activity of YAP and TAZ is necessary to direct proper levels of *Sox9* expression and to determine Sertoli cell fate. For instance, basal levels of YAP and TAZ expression may be necessary for proper expression of SOX9 in the developing Sertoli cells, while overexpression of YAP and TAZ could lead to its inactivation. Indeed, a phenomenon similar to this has been reported to occur in the developing kidney, where the deletion of *Yap* in the cap mesenchyme prevents nephron formation [63], whereas the overexpression of YAP/TAZ leads to the differentiation of nephron progenitors into myofibroblasts [64].

Aside from the Sertoli cells, the interstitial tissues were also affected in the *Lats1*^flox/flox^;*Lats2*^flox/flox^;*Nr5a1*-cre animals. Loss of steroidogenic FLCs and the concomitant appearance of a spindle-shaped cell population leading to the development of interstitial fibrosis was observed in the testis of mutant animals at e16.5. Because steroidogenic cells progressively transdifferentiate into myofibroblast-like cells in the adrenal cortex of *Lats1*^flox/flox^;*Lats2*^flox/flox^;*Nr5a1*-cre animals [42], we hypothesized that FLCs (which share a common origin with the steroidogenic cells of the adrenal cortex) also transdifferentiate into myofibroblast-like cells in these animals. Interestingly, it was previously shown that disruption of *Nr5a1* in FLCs leads to massive interstitial fibrosis caused by the synthesis of extracellular matrix by the *Nr5a1*-disrupted FLCs [65], suggesting that loss of *Nr5a1* in our model could be responsible for the observed fibrosis. Though it seems likely that some myofibroblast-like cells were of Leydig cell origin, we cannot rule out the possibility that others originated from peritubular myoid cells or other stromal cell types. Lineage tracing experiments would be needed to determine their origin unequivocally. Hippo signaling inactivation and YAP/TAZ transcriptional activation have also been shown to play a major roles in the transdifferentiation of cells into myofibroblasts and fibrosis in non-endocrine tissues such as the lung [66] and the heart [67] as well as in tissues derived from the intermediate mesoderm such as the kidney [68] and the Mullerian duct [69]. Among the known downstream targets of YAP/TAZ, CTGF could play an important role in the transdifferentiation of FLCs into myofibroblast-like cells. Indeed, CTGF has been previously described as a key driver of fibrosis [70,71,72,73] and myofibroblast formation [74,75,76]. Furthermore, it was shown that TAZ was dramatically enriched on the promoter of *Ctgf* following inactivation of *Lats1/2* in mouse Mullerian ducts, leading to *Ctgf* overexpression and myofibroblast transdifferentiation [69]. 

Even though we attributed the phenotypic changes observed in the Sertoli cells and FLCs of *Lats1*^flox/flox^;*Lats2*^flox/flox^;*Nr5a1*-cre mice to the direct loss of *Lats1/2* in these cell types, there is also a possibility that the loss of *Lats1/2* in Sertoli cells affected the FLCs and vice versa. For example, loss of DHH secretion by Sertoli cells in *Lats1*^flox/flox^;*Lats2*^flox/flox^;*Nr5a1*-cre animals could affect FLC differentiation [11]. Conversely, loss of Activin A secretion by FLCs could indirectly affect Sertoli cells and testis cord formation [77]. Activin A increases SOX9 nuclear localization in the esophageal adenocarcinoma cell line FLO-1 [78], suggesting that Activin A could also regulate SOX9 in the Sertoli cells. Furthermore, transdifferentiation of the FLC population into myofibroblast-like cells could mechanically interfere with testis cord development. In agreement with this hypothesis, it was shown that inactivation of *Lats1*/*2* in the Müllerian duct mesenchyme cells prior to the degradation of the Müllerian ducts in males leads to their differentiation into myofibroblasts, which in turn interfere with the development and the coiling of the adjacent epididymides [69]. Transgenic models that target *Lats1*/*2* specifically in the Sertoli cells and the FLCs are needed to distinguish between the direct and indirect effects in each cell population. Lastly, though an increase in YAP/TAZ transcriptional activity in the testes of *Lats1*^flox/flox^;*Lats2*^flox/flox^;*Nr5a1*-cre animals is the presumptive driving force behind the observed phenotypic changes, it is important to mention that LATS1/2 can occasionally act independently of the canonical Hippo pathway [79,80]. The generation of a *Lats1*^flox/flox^;*Lats2*^flox/flox^;*Yap*^floxflox^;*Taz*^floxflox^;*Nr5a1*-cre mouse model is needed to determine if YAP/TAZ stabilization is solely responsible for the effects of *Lats1/2* loss.

In summary, we report here a previously unheard role of the Hippo pathway in the development of the testis, as loss of *Lats1/2* results in testis cord dysgenesis and interstitial fibrosis. Further studies are required to define the mechanism of action of Hippo signaling throughout testis development in both FLC and Sertoli cell differentiation.

## 4. Material and Methods

### 4.1. Ethics

Animal procedures were approved by the Comité d’Éthique de l’Utilisation des Animaux of the Université de Montréal (protocol numbers Rech-1739 and Rech-1909 respectively approved in 2015 and 2017) and conformed to the guidelines of the Canadian Council on Animal Care. 

### 4.2. Transgenic Mouse Strains

*Nr5a1*-cre mice (FVB-Tg-*Nr5a1*^Cre7Lowl/J^) were obtained from the Jackson Laboratory and maintained by crossing Cre-positive males with wild-type females (C57BL/6J). *Lats1*^flox/flox^ (*Lats1*^tm1.1JFm/RjoJ^) and *Lats2*^flox/flox^ (*Lats2*^tm1.1JFm/RjoJ^) mice were obtained from Dr. Randy L. Johnson (M.D. Anderson Cancer Center, Houston, TX, USA). Mice were selectively bred over several generations to obtain the *Lats1*^flox/flox^;*Nr5a1*-cre, *Lats2*^flox/flox^;*Nr5a1*-cre and *Lats1*^flox/flox^;*Lats2*^flox/flox^;*Nr5a1*-cre genotypes. Genotype analyses were done on tail biopsies by PCR as previously described for Cre [41] and *Lats1/2* [81].

### 4.3. Tissue Collection

All embryos or testes were collected from e12.5 to e17.5, 1dpp (day post-partum) and 2-week-old *Lats1*^flox/flox^;*Lats2*^flox/flox^;*Nr5a1*-cre male mice and *Lats1*^flox/flox^;*Lats2*^flox/flox^ control littermates and were fixed in 4% paraformaldehyde for 4 h (whole embryos, 1 dpp testes) or Bouin’s fixative overnight (testes from 2-week-old mice) and embedded in paraffin for histopathologic analyses or immunohistochemistry (IHC). Some testes from e14.5, e17.5, and 1dpp animals were flash frozen followed by homogenization for quantitative RT-PCR (RT-qPCR).

### 4.4. Histopathology and Immunohistochemistry 

Histopathology analyses were performed on paraffin embedded, 5 μm thick tissues stained with hematoxylin and eosin (H&E) or Masson’s trichrome. Immunohistochemistry analyses were performed on paraffin-embedded, 5 μm thick tissue sections using VectaStain Elite avidin–biotin complex method kits (Vector Laboratories, Newark, CA, USA) or mouse on mouse (M.O.M.) elite peroxidase kit (Vector Laboratories) as directed by the manufacturers. Sections were probed with antibodies against cCASP3, CYP17A1, DDX4, GJA1, α-SMA, SOX9, TAZ, VIM, WT1, YAP, or phospho-YAP. Staining was done using the 3,3′-diaminobenzidine peroxidase substrate kit (Vector Laboratories) or with a chromogen (Agilent) and counterstained with hematoxylin before mounting. Negative controls consisted of slides for which the primary antibody was omitted. Antibodies used are listed in Supplemental Table S1. 

### 4.5. Reverse-Transcription-Quantitative PCR

Total RNA from testes of e14.5, e17.5, and 1 dpp animals was extracted using the Total RNA Mini Kit (FroggaBio, Concord, ON, Canada) according to the manufacturer’s protocol. Total RNA was reverse-transcribed using 100 ng of RNA and the SuperScriptVilo™ cDNA synthesis kit (Thermo Fisher scientific, Waltham, Ma, U.S.). Real-time PCR reactions were run on a CFX96 Touch instrument (Bio-Rad, Hercules, CA, USA), using Supergreen Advanced qPCR MasterMix (Wisent, St-Bruno, Qc, Canada). Each PCR reaction consisted of 7.5 μL of Power SYBR Green PCR Master Mix, 2.3 μL of water, 4 μL of cDNA sample and 0.6 μL (400 nmol) of gene-specific primers. PCR reactions run without cDNA (water blank) served as negative controls. A common thermal cycling program (3 min at 95 °C, 40 cycles of 15 s at 95 °C, 30 s at 60 °C and 30 s at 72°C) was used to amplify each transcript. To quantify relative gene expression, the Ct of genes of interest was compared with that of *Rpl19*, according to the ratio R = [E^Ct *Rpl19*^/E^Ct target^] where E is the amplification efficiency for each primer pair. *Rpl19* Ct values did not change significantly between tissues, and *Rpl19* was therefore deemed suitable as an internal reference gene. The specific primer sequences used are listed in Supplemental Table S2.

### 4.6. Statistical Analyses

All statistical analyses were performed with Prism software version 6.0d (GraphPad Software Inc., CA, USA, RRID: SCR_002798). All the datasets were subjected to the *F* test to determine the equality of variances. Student’s *t*-test was used for all comparisons between genotypes. Means were considered significantly different when *p* value was < 0.05. All data are presented as means ± SEM.

## Data Availability

All relevant data are within the paper and its supporting information files.

## Supplementary Materials

The following supporting information can be downloaded at: https://www.mdpi.com/article/10.3390/ijms232113585/s1.
