# Supplementary material for: Targeted Disruption of Lats1 and Lats2 in Mice Impairs Testis Development and Alters Somatic Cell Fate
Source: Int J Mol Sci. 2022 Nov 5;23(21):13585. doi: 10.3390/ijms232113585 (PMC9655313; doi:10.3390/ijms232113585)
Supplement: Supplementary file 1 [file ijms-23-13585-s001.zip › ijms-1978618-supplementary.pdf]

Supplemental data

Table S1. List of Antibodies

Antibody	Company	Catalog number (and RRID)	Dilution
**cCASP3**	Cell Signaling	9661, (RRID : AB_2341188)	1/50
**CYP17A1**	Santa Cruz	sc-46081, (RRID : AB_2088659)	1/100
**DDX4**	R&D Sytems	AF2030, (RRID: AB_2277369)	1/200
**GJA1**	Cell Signaling	3512, (RRID: AB_2294590)	1/100
**α-SMA**	BioGenex	MU128-UC, (RRID : AB_2335623)	1/100
**SOX9**	Cell Signaling	82630, (RRID: AB_2665492)	1/250
**TAZ**	Sigma-Aldrich	HPA007415, (RRID : AB_1080602)	1/500
**VIM**	Cell signaling	5741, (RRID: AB_10695459)	1/100
**WT1**	Cell signaling	83535, (RRID: AB_2800020)	1/100
**YAP**	Cell signaling	14074, (RRID: AB_2650491)	1/300
**pYAP**	Cell Signaling	13008, (RRID: AB_2650553)	1/100

Table S2. Quantitative RT-qPCR primer sequences

Gene	Forward	Reverse
Acta2	AGCCATCTTTCATTGGGATGG	CCCCTGACAGGACCTTGTTA
Ankrd1	GGATGTGCCGAGGTTTCTGA	GCCGTCCGTTTATACTCATCG
Cald1	GTTGCTGCCCTAGAGATAGTCA	AACCTTTGACTGTCCACCCC
Ctgf	GAGGAAAACATTAAGAAGGGCAAAA	CCGCAGAACTTAGCCCTGTA
Cyp17a1	GGTGGACATATTCCCGTGGTT	CTGGCCTTCCCCAGTGTTTT2
Cyp26b1	GCAAGATCCTACTGGGCGAA	TTGGAGAAGACCTTGCGCTT
Cyr61	TTGACCAGACTGGCGCTCT	AGTTTTGCTGCAGTCCTCGT
Dhh	CGCCTGATGACAGAGCGTT	AGTGGAGTGAATCCTGTGC
Dmrt1	TGGCAGATGAAGACCTCAGAGAG	CGAGAACACACTGGCTTTGGC

Foxl2	ACAACACCGGAGAAACCAGAC	CGTAGAACGGGAACTTGGCTA
Gli1	CCTGCCAGCTGAAGTCAGAG	AACAGGTGATCCTGTGTGCC
Hsd3b1	AGCTGCAGACAAAGACCAAGGTGA	GAACACAGGCCTCCAATAGGTTCT
Lats1	AGCAGCACGTAGAGAACGTC	TCTCATTTGATCCTGGGCATCT
Lats2	TGCACTGGATTCAGGTGGACTCA	GAGAATGTGCCAGGCACCTCT
Nr5a1	TCTCTAACCGCACCATCA	TCGACAATGGAGATAAAGGT
Ocln	AAGTCAACACCTCTGGTGCG	TCCTGCAGACCTGCATCAAA
Pard6b	GTTTCTCCTCTCACACGGCACCCA	GGACCTCCACAGCCAAGACTACT
Ptch1	ACGGCCGGGATCATTGTCAT	CGGTGAACTCCACTCCGATG
Pdfra	AGAGGCAAGATCCCTGGCTA	CCATAGCTCCTGAGACCTGC
Rpl19	CTGAAGGTCAAAGGGAATGTG	GGACAGAGTCTTGATGATCTC
Sox9	AGGAAGTCGGTGAAGAACGG	GGACCCTGAGATTGCCCAGA
Star	GATTAAGGCACCAAGCTGTGCTG	CTGCTGGCTTTCCTTCTTCCAGC
Spp1	CCTTGCTTGGGTTTGCAGTC	TGGTCGTAGTTAGTCCCTCAGA
Wnt4	AGCTGTCATCGGTGGGCAGCAT	ACTGTCCGGTCACAGCCACACT
Wt1	AGCTGTCCCACTTACAGATGCAT	GGATGCTGGACTGTCTCCGTGT

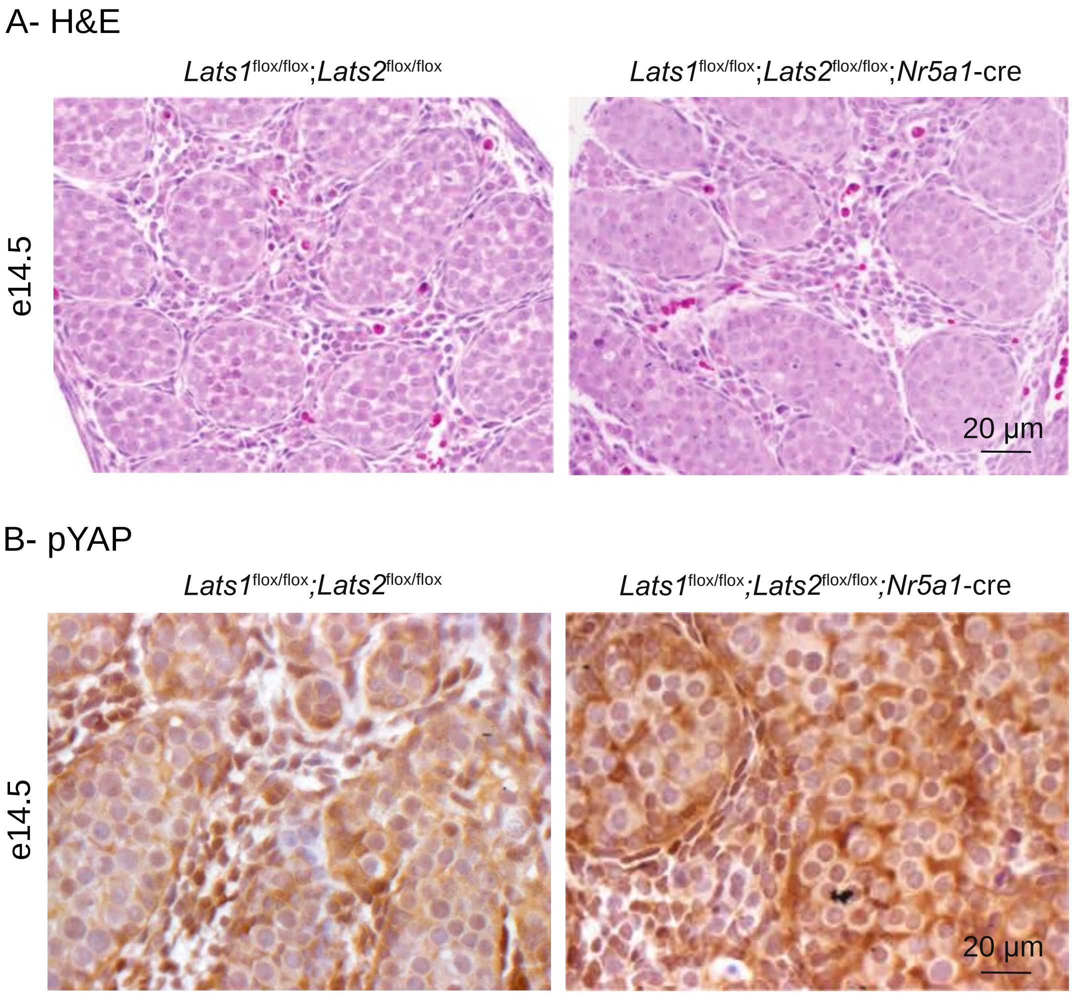

Figure S1. Absence of abnormal phenotype in the testes of some e14.5 *Lats1*$^{flox/flox}$;*Lats2*$^{flox/flox}$;*Nr5a1*-cre. (**A**) Photomicrographs comparing testis histology of *Lats1*$^{flox/flox}$;*Lats2*$^{flox/flox}$;*Nr5a1*-cre (with inefficient recombination or before recombination occurs) with that of *Lats1*$^{flox/flox}$;*Lats2*$^{flox/flox}$ controls. Scale bar (lower right) is valid for all images. Hematoxylin and eosin stain. (**B**) Immunohistochemical analysis of phospho-YAP expression in the testes of e14.5 mice of the indicated genotypes showing the absence of Hippo signaling inactivation in some *Lats1*$^{flox/flox}$;*Lats2*$^{flox/flox}$;*Nr5a1*-cre. Scale bar in (lower right) is valid for all images.

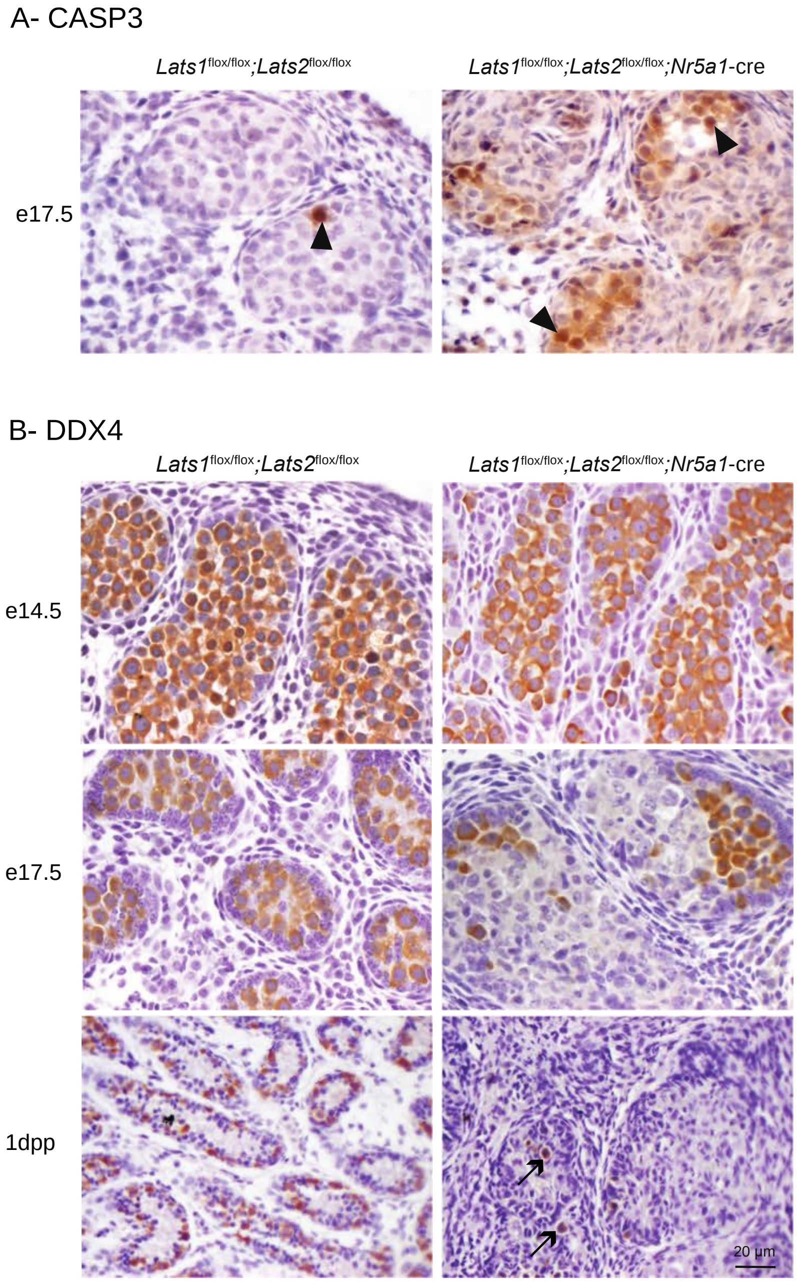

Figure S2. Progressive loss of germ cells in the testis cords of *Lats1*$^{flox/flox}$;*Lats2*$^{flox/flox}$;*Nr5a1*$^{cre/+}$ mice. (**A**) Immunohistochemical analysis of cCASP3 expression in testes of mice of the indicated genotypes. Scale bar in the right panel is valid for the left panel. (**B**) Immunohistochemical analysis of DDX4 expression in testes of mice of the indicated genotypes. Arrowhead = apoptotic germ cells. Arrow = remaining germ cells. Scale bar (lower right) is valid for all images.

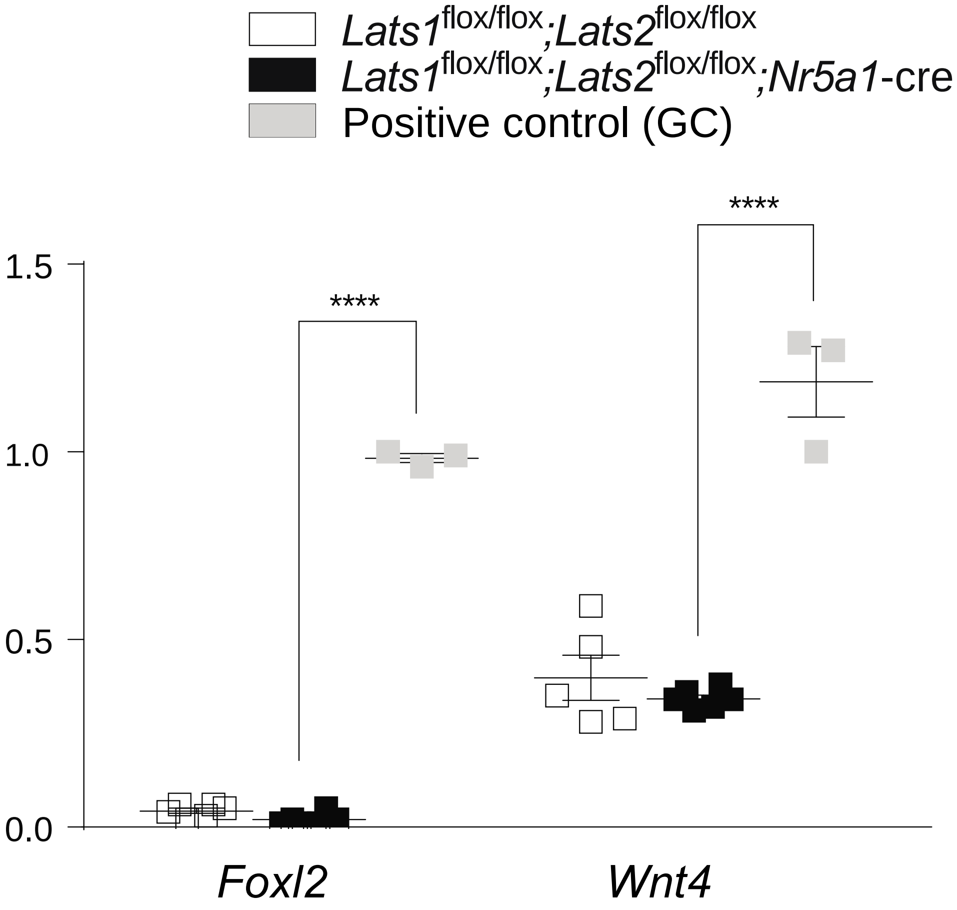

Figure S3. Sertoli cells of *Lats1*$^{flox/flox}$;*Lats2*$^{flox/flox}$;*Nr5a1*-cre mice do not transdifferentiate in granulosa cells. RT-qPCR analysis of the female markers *Foxl2* and *Wnt4* in testes of e17.5 mice of the indicated phenotype (n = 5 or 6) compared to expression in control granulosa cells (n = 3). All data were normalized to the housekeeping gene Rpl19 and are expressed as means (columns) ± SEM (error bars). GC = granulosa cells, Asterisks = significantly different from control (**** P< 0.0001).
